# ABHD1 Facilitates Intermediate Filament–Mediated Endothelial Cell Chemotaxis by Regulating KRT1 and KRT2 in Diabetic Retinopathy

**DOI:** 10.1155/jdr/5513165

**Published:** 2024-11-20

**Authors:** Xinyi Liu, Junwei Fang, Tian Niu, Xindan Xing, Xin Shi, Yu Xiao, Yuan Qu, Yan Jiang, Kangjia Lv, Tianyu Dou, Qian Zhu, Hancong Wan, Xiaoxin Liu, Hanying Wang, Kun Liu

**Affiliations:** ^1^Department of Ophthalmology, Shanghai General Hospital, Shanghai Jiao Tong University School of Medicine, Shanghai 200080, China; ^2^National Clinical Research Center for Eye Diseases, Shanghai General Hospital, Shanghai Jiao Tong University School of Medicine, Shanghai 200080, China; ^3^Shanghai Key Laboratory of Ocular Fundus Diseases, Shanghai 200080, China; ^4^Shanghai Engineering Center for Visual Science and Photomedicine, Shanghai 200080, China; ^5^Shanghai Engineering Center for Precise Diagnosis and Treatment of Eye Diseases, Shanghai 200080, China

**Keywords:** ABHD1, diabetic retinopathy, endothelial cell chemotaxis, intermediate filament, keratin 1, keratin 2

## Abstract

Diabetic retinopathy (DR) is one of the most common complications of diabetes and induces severe visual impairment worldwide. Endothelial cell dysfunction plays an important role in the pathogenesis of DR. Here, we keep a watchful eye on *α*/*β*-hydrolase domain–containing 1 (ABHD1), a potential regulator in lipid metabolism and neovascularization. Results revealed that ABHD1 expression increased both in retina tissues of DR patients and in high-glucose–treated human retina endothelial cells. Inhibition of ABHD1 remitted endothelial cell proliferation and migration. And GSEA uncovered that ABHD1 knockdown remits endothelial cell chemotaxis and intermediate filament (IF) might be mediated in the progress by regulating keratin 1 (KRT1) and keratin 2 (KRT2). Therefore, we assume that ABHD1 is concerned with endothelial cell proliferation and migration in DR, consequently leading to pathological neovascularization. The findings may provide a potential therapeutic target for DR.

## 1. Introduction

Diabetic retinopathy (DR) is one of the most prevalent microvascular complications of diabetes and acts as the main cause of visual impairment and blindness in the working population [1, 2]. Current multimodality treatments for DR include laser photocoagulation, intravitreous injections of anti-VEGF substance, and vitrectomy [3]. However, refractory vision-threatening DR still shows poor response to conventional therapy. Accordingly, exploring novel molecular mechanisms in DR is imperative to preserve DR patient's visual function.

The pathogenesis of DR is a multifactorial process concerning various components of the retina. Lipid metabolism dysregulation has been proved to be associated with DR progression. Elevated low-density lipoprotein (LDL) level increases the incidence of retinal hard exudation [4]. Under pathological condition, LDL extravasation has cytotoxicity to retinal endothelial cells, pericytes, and Müller cells, which then result in retinal damage of DR [5–7]. Furthermore, DR is increasingly deemed as a chronic low-grade inflammation disease with elevated level of various cell-derived inflammatory cytokines and growth factors [8–10]. Under a diabetic state, glucose dysregulation and lipid metabolism prompt chronic inflammation with increasing levels of inflammatory biomarkers [11]. Inflammatory manifestations, for instance, leukocyte stasis and increased leukocyte–endothelial cell adhesion, occur in the retinal capillary of DR patients, thus leading to permanent capillary occlusion presented as acellular capillaries in DR retina [9, 12]. Additionally, oxidative stress is also regarded as a vital causative factor in the pathogenesis of DR marked by excessive accumulation of reactive oxygen (ROS) [13, 14]. In recent years, research has revealed that lipid peroxidation is concerned with the pathogenesis of DR. Lipid peroxidation and advanced lipoxidation end product (ALE) formation are considered as key factors in the development of DR [15]. In the streptozotocin (STZ)-induced rat model, lipid peroxidation products in blood were reported to be related with rat retina changes [11, 16].

*α*/*β*-Hydrolase domain–containing 1 (ABHD1) is a 43-kDa protein from the ABHD family, which appears to become a potential regulator in lipid metabolism [17]. The biochemical function of ABHD1 has rarely been explored. In renal cell lines, overexpression of ABHD1 is related with ROS production, and in oxidative stress–induced hypertension mouse model, kidney ABHD1 expression significantly ascends [18]. Lyso-phosphatidylcholine (LPC) is a kind of proinflammatory lipid originating from degradation products of phosphatidylcholine and can promote the generation of inflammatory factors [19, 20]. LPC expression increases in the erythrocyte membrane of diabetic patients, especially those diagnosed with DR [21]. The levels of LPC in plasma LDL of diabetic patients are significantly higher than those of healthy controls especially in proliferative DR patients [22]. Hepatic ABHD1 exhibits significant negative correlation with plasma LPC, suggesting that ABHD1 is involved in the regulation of plasma LPC and inflammation response [23]. Another large-scale genome-lipid research also proves that plasma LPC 14:0 has a strong correlation with ABHD1, emphasizing the tight relation between LPC and ABHD1 [24].

Intriguingly, in consideration that ABHD1 has compact correlation with dyslipidemia, inflammation response, and oxidative stress, whether ABHD1 acts as a latent pathogenic factor in DR had drawn our attention. This research is aimed at investigating the function of ABHD1 and its potential effects on DR progression. The findings indicate that ABHD1 may be a novel treatment factor implicated in DR progression.

## 2. Method

### 2.1. Cell Culture and Treatment

Human umbilical vein endothelial cells (HUVECs) were purchased from Cell Systems (Kirkland, Washington, United States) and cultured in endothelial cell culture medium (ECM, ScienCell, California, United States); the medium was supplemented with 5% fetal bovine serum (FBS), 1% penicillin/streptomycin solution, and 1% endothelial cell growth supplement (ECGS). The cell culture environment was maintained at 37°C, and the air contained 5% CO_2_.

Human retina microvascular endothelial cells (HRMECs) were purchased from Thousand (Shanghai, China) and cultured in ECM (ScienCell, CA, USA); the medium was supplemented with 10% FBS and 1% penicillin/streptomycin solution. HRECs were treated with 25 mmol/L glucose (HG) (D-glucose, Sigma, America) for 24 h, 48 h, and 72 h, respectively, and the control group was treated with 25 mmol/L mannitol (NG) (D-mannitol, Sigma, America) as osmotic control. The cell culture environment was maintained at 37°C, and the air contained 5% CO_2_.

### 2.2. Cell Transfection

siRNA for ABHD1 (siABHD1-555, siABHD1-680) and siRNA-negative control (siNC) were both purchased from GenePharma (Pudong, Shanghai, China). And overexpression plasmid for ABHD1 (H39090) and control plasmid (GL107) were purchased from Obio (Shanghai, China). When the fusion degree of HUVECs reached about 60%, transfection was performed using Lipofectamine RNAiMAX transfection reagent (Invitrogen, Carlsbad, California, United States) according to the reagent instructions at siRNA concentration of 5 *μ*g/*μ*L and Lipofectamine 3000 transfection reagent (Invitrogen, Carlsbad, California, United States) according to instructions at plasmid concentration of 2.5 *μ*g/*μ*L. After incubation for 48 h, the transfection efficiency was verified by real-time fluorescence quantitative polymerase chain reaction (qRT-PCR) and Western blot polymerase chain reaction. The transfected cells will be used in the following experiments.

### 2.3. Real-Time Quantitative PCR

Total RNA was isolated from HUVECs using the TRIzol (Invitrogen, Carlsbad, California, United States) kit according to the instructions. The extracted RNA was reverse transcribed with the HiScript 1st strand cDNA synthesis kit of Vazyme (China). qRT-PCR was performed using ACEQ qPCR SYBR Green Master Mix (Vazyme, China), and the procedure was performed according to the manufacturer's instructions for an ABI Prism 7500 sequence detection system (Applied Biosystems, Foster City, California, United States). Each sample was repeated three times, and the values of ABHD1, keratin 1 (KRT1), and keratin 2 (KRT2) were calculated using the 2-*ΔΔ*CT method, which was unitized using 18S rRNA cyclic number. The primer sequences for each cDNA are shown in Table 1.

### 2.4. Western Blot

Cells were collected and protease inhibitor in RIPA lysate with phenylmethanesulfonyl fluoride (PMSF). The samples were separated by SDS-PAGE and transferred to a nitrocellulose membrane. Blots were incubated with specific primary antibodies (anti-ABHD1, Proteintech, 1 : 1000 dilution; anti-cytokeratin 1, Abcam, 1 : 2000 dilution; anti-KRT2, Absin, 1 : 2000 dilution; *β*-actin, Abclonal, 1 : 20,000 dilution) overnight at 4°C and then with secondary antibody (anti-rabbit IgG, Cell Signaling Technology, 1 : 5000 dilution) for 1 h at room temperature. Protein bands were colored using a SuperSignal ECL detection kit (Thermo Fisher Scientific, United States), and exposed proteins were captured using a ChemiDoc imaging system (Bio-Rad). Protein quantitative analysis was performed using ImageJ 1.53 K software.

### 2.5. Cell Scratch Assay

HUVECs were seeded in 12-well plates at a cell density of 5.0 × 10^5^ cells/mL. After 48 h of grouping treatment, serum starvation was performed for 24 h when the confluence of HUVECs reached approximately 100%. Then, a 200-mL sterilized pipette tip was used to scratch the monolayer fusion cells in the center of the culture hole to form a scratch. The cells were washed with phosphate-buffered saline (PBS) and cultured in serum-free medium. The migration areas in each plate were observed by an Olympus (Tokyo, Japan) microscope and photographed at 0, 24, and 48 h, respectively, and quantitatively analyzed by ImageJ 1.53 K software [25, 26].

### 2.6. Cell Counting Kit 8 (CCK-8) Cell Proliferation Experiments

The log-phase HUVECs were seeded in 96-well plates at a cell density of 5.0 × 10^4^ cells/mL. For cell proliferation assays, a CCK-8 (Dojindo Laboratory, Shanghai, China) was used and operated according to the manufacturer's instructions. After 24 h of treatment as prescribed, 10 mL of CCK-8 solution was added to the wells, incubated at 37°C for 4 h, and then, optical density (OD) values were measured at 450 nm wavelength with an Infinite microplate reader (Tecan, Männedorf, Switzerland).

### 2.7. Statistical Analysis

The possible biological processes of ABHD1 were analyzed by gene set enrichment analysis (GSEA) software V4.1.0. The enrichment score was calculated by using molecular signature database (https://http://ww.gsea-msigdb.org/gsea/MSIGDB) V 7. The *p* value was calculated by 1000 permutations.

The cluster profiler program was used to perform Gene Ontology (GO) functional enrichment analysis pathway enrichment, with *p* < 0.05 as significant enrichment criterion. The heat map and violin diagram of gene function analysis were generated by R software.

Statistical analyses were performed using Prism 9 (GraphPad, United States) software. Two-tailed unpaired *t*-tests were used to compare two sets of data. One-way ANOVA and two-way ANOVA were used for multiple comparisons. Data are shown as mean ± SD. The *p* values in the experimental results are expressed as follows: ⁣^∗^*p* < 0.05, ⁣^∗∗^*p* < 0.01, ⁣^∗∗∗^*p* < 0.001, ⁣^∗∗∗∗^*p* < 0.0001, and NS for difference not significant.

## 3. Result

### 3.1. ABHD1 Increased in DR Patient Retina as DR Progressed and Increased in High-Glucose–Treated Endothelial Cells In Vitro

**Figure 1:**
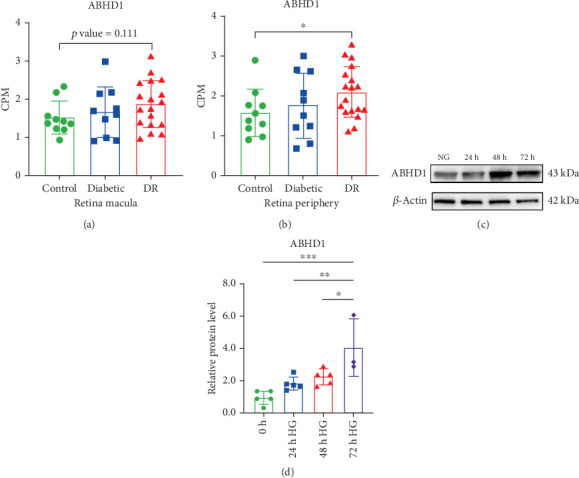
ABHD1 increased in the macular and peripheral retina in DR and in HG-treated HRECs. (a) Counts per million (CPM) of ABHD1 expression in the peripheral retina (*n* = 10 replicates). (b) Counts per million (CPM) of ABHD1 expression in the macula of the retina (*n* = 10 replicates). (c, d) Expression of ABHD1 in HG-treated HRECs after 24, 48, and 72 h, respectively (*n* = 5 replicates). ⁣^∗^*p* < 0.05; ⁣^∗∗^*p* < 0.01.

In order to endorse ABHD1 expression change in the retina of DR patients, we downloaded transcriptome raw data (GSE160306) provide by Becker et al. from the GEO database. The database contains 38 RNA samples derived from the retinas of 38 patients, comprising 10 healthy controls, 10 diabetic patients without retinopathy, 16 NPDR patients, and 2 PDR patients. Data analysis revealed that ABHD1 mRNA expression levels increased in both peripheral and macular regions of the retina as DR severity progressed, and ABHD1 mRNA expression levels in NPDR patients were significantly higher than those in healthy controls (Figures 1(a) and 1(b)). Analogously, HRMECs were treated with high glucose to discriminate ABHD1 fluctuation in a high-glucose environment. After 24, 48, and 72 h high-glucose exposure, ABHD1 expression presented an evident rise in HRMECs (Figures 1(c) and 1(d)).

### 3.2. ABHD1 Was Positively Correlated With Endothelial Cell Chemotaxis

**Figure 2:**
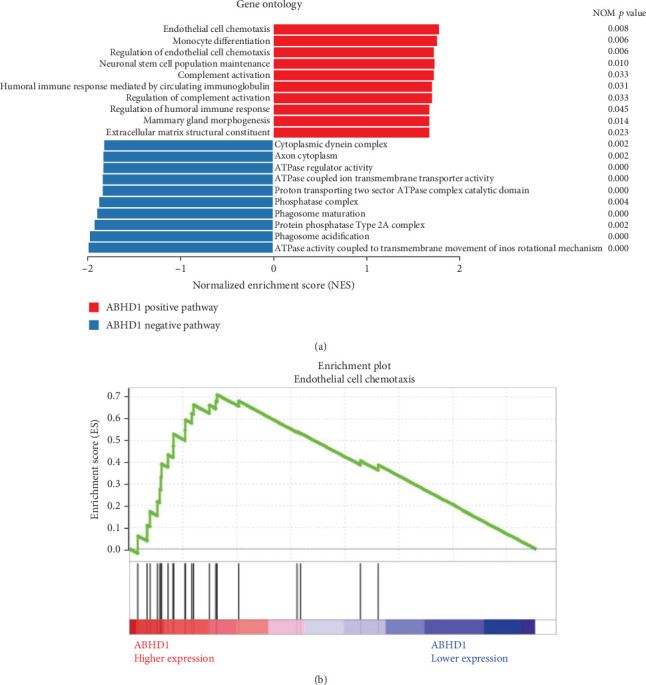
ABHD1 was positively correlated with endothelial cell chemotaxis. (a) Gene Ontology of the first 10 pathways showed positive correlation (red) and negative correlation (blue) with ABHD1 expression, respectively. (b) GSEA showed the elevated expression of ABHD1 was closely related to the chemotaxis of endothelial cells.

To further determine the related phenotype and biological pathways of ABHD1 involved in DR pathogenesis, Gene Oncology (GO) analysis and GSEA were performed on the GSE160306 data set. Top 10 pathways of positive and negative correlations with ABHD1 expression were exhibited, respectively, according to the pertinency with ABHD1 expression (Figure 2(a)). Thereinto, endothelial cell chemotaxis was closely related to the expression of ABHD1 (Figure 2(b)).

### 3.3. Inhibition of ABHD1 Mitigated Endothelial Cell Proliferation and Migration

**Figure 3:**
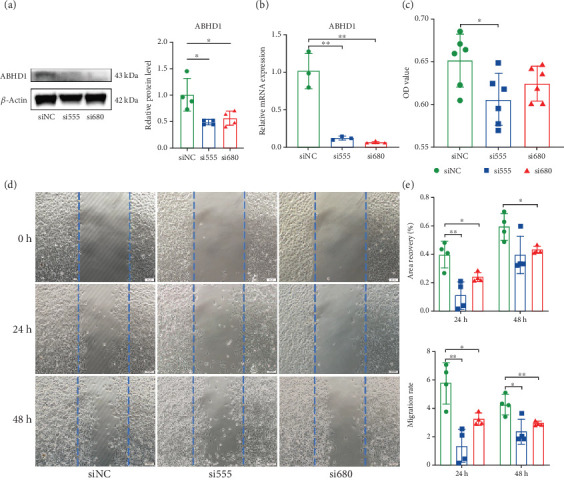
Inhibition of ABHD1 mitigated endothelial cell proliferation and migration. (a) Western blot was used to detect the translation level of ABHD1 inhibition (*n* = 4 replicates). (b) qPCR was used to detect the transcription level of ABHD1 inhibition (*n* = 3 replicates). (c) The proliferation of endothelial cells in si555, si680, and siNC groups was detected by CCK-8 (*n* = 6 replicates). (d, e) The migration of endothelial cells in si555, si680, and siNC groups was detected by scratch test (*n* = 4 replicates). ⁣^∗^*p* < 0.05; ⁣^∗∗^*p* < 0.01.

To clarify the relationship between ABHD1 and pathological changes in endothelial cells, we targetedly knocked down ABHD1 with siRNA and confirmed the knockdown level by qPCR and Western blot (Figures 3(a) and 3(b)). CCK-8 results showed that the proliferation level of HUVECs in the ABHD1 knockdown group was significantly lower than that in the siNC group (Figure 3(c)). Wound healing experiments showed that ABHD1 promoted migration of HUVECs (Figures 3(d) and 3(e)).

### 3.4. ABHD1 Facilitated Intermediate Filament (IF)–Mediated Endothelial Cell Chemotaxis by Regulating KRT1 and KRT2

**Figure 4:**
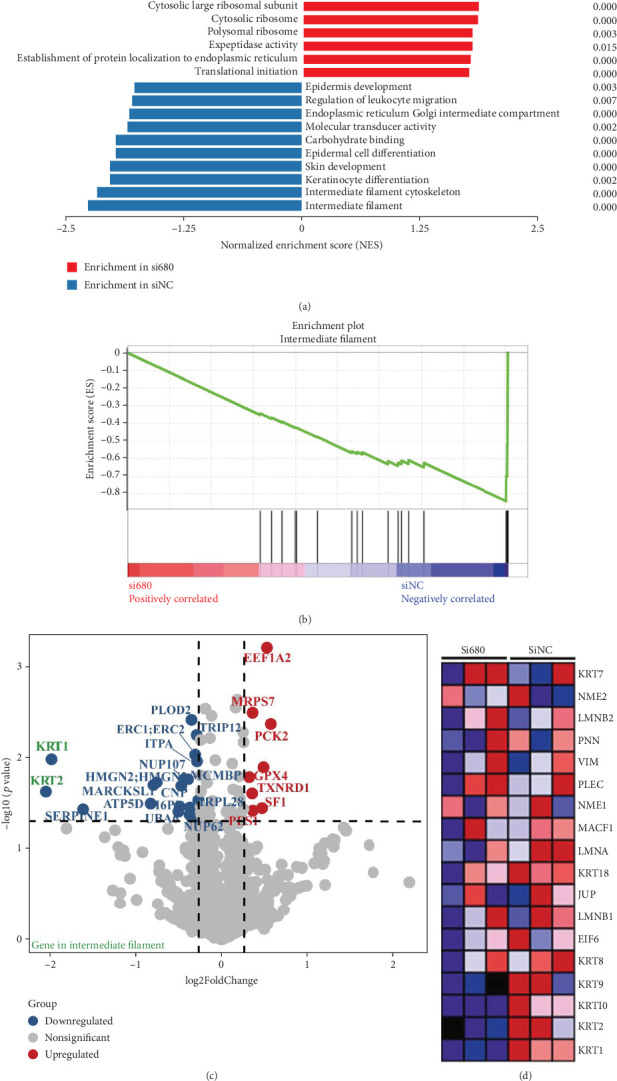
ABHD1 facilitated intermediate filament (IF)–mediated endothelial cell chemotaxis by regulating KRT1 and KRT2. (a, b) Gene Ontology and GSEA inhibited IF was identified as the most inhibited pathway. (c) The volcano plot showed the differential proteins between the si680-treated group and the control group. Blue and red dots denoted downregulated and upregulated proteins, respectively (FC > 1.2, *p* < 0.05). (d) Heatmap presented enriched IF pathway–related proteins.

**Figure 5:**
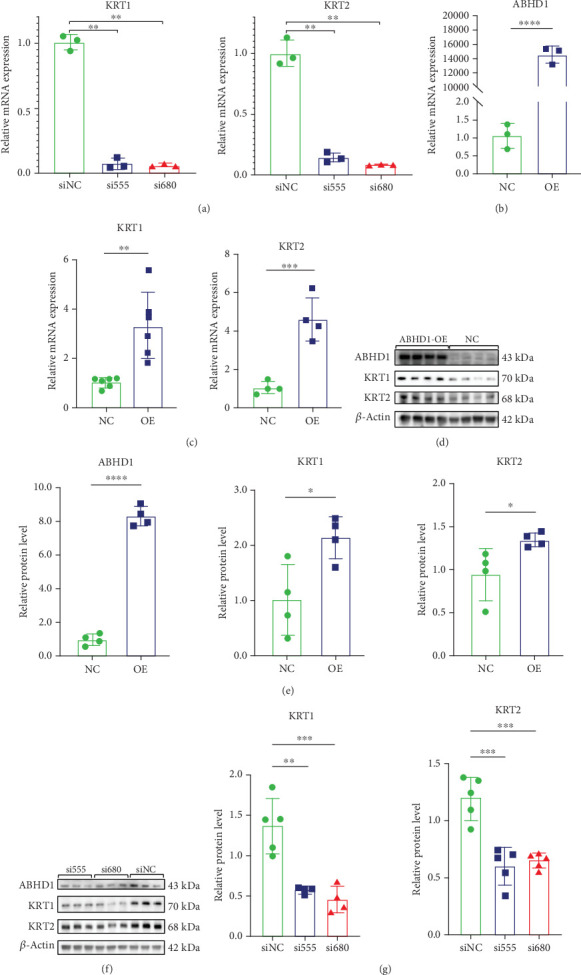
(a) qPCR was used to detect the transcription level of KRT1 and KRT2 in the si555 and si680 groups compared with the siNC group (*n* = 3 replicates). (b) mRNA level of the ABHD1 overexpression group and the control group (*n* = 3 replicates). (c) qPCR was used to detect the transcription level of KRT1 (*n* = 6 replicates) and KRT2 (*n* = 4 replicates) in the ABHD1 overexpression group and the control group. (d, e) Western blot showed the protein expression of ABHD1, KRT1, and KRT2 in the ABHD1 overexpression group and the control group (*n* = 4 replicates). (f, g) Western blot showed the protein expression of ABHD1, KRT1, and KRT2 in the si555 and si680 groups compared with the siNC group (*n* = 5 replicates). ⁣^∗^*p* < 0.05; ⁣^∗∗^*p* < 0.01.

So as to dig out involved pathways of ABHD1 in endothelial cell chemotaxis, most enriched pathways were identified by GO and GSEA in ABHD1-knockdown HUVECs using siRNA. At the core of results, the IF-related pathway was the most repressed pathway, accordingly manifesting that IF-associated proteins may act as direct or indirect targets of ABHD1 in regulating endothelial cell chemotaxis (Figures 4(a) and 4(b)). In endothelial cells with low ABHD1 expression, the expression levels of IF-related proteins KRT1 and KRT2 were significantly reduced (Figures 4(c) and 4(d)). qPCR and Western blot revealed that the mRNA and protein level of KRT1 and KRT2 increased after overexpressing ABHD1 (Figures 5(a), 5(f), and 5(g)) while it decreased by knocking down ABHD1 (Figures 5(b), 5(c), 5(d), and 5(e)). According to these results, ABHD1 might mediate endothelial cell chemotaxis by regulating KRT1 and KRT2.

## 4. Discussion

**Figure 6:**
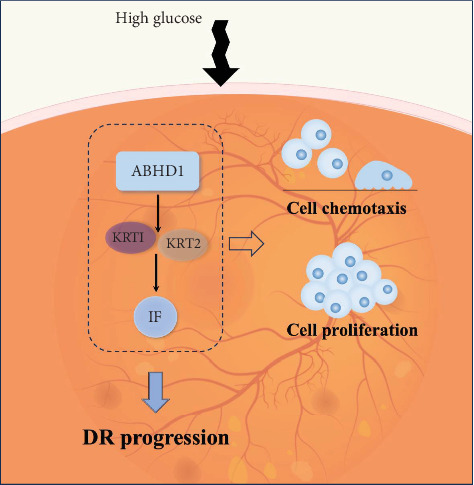
Mechanistic diagram illustrates how ABHD1 influences DR.

In this research, we found that ABHD1 level increased under hyperglycemic status and ABHD1 knockout remitted endothelial cell chemotaxis. The regulation of endothelial chemotaxis induced by ABHD1 in a high-glucose environment may be mediated by KRT1 and KRT2 (Figure 6).

Proliferative DR, as advanced stage of DR, is marked by retinal pathological neovascularization and always leads to severe complications, preretinal fibrovascular membranes and vitreous hemorrhage, for instance [27]. Under hypoxia and hyperglycemic environment, retinal tissue upregulates factors associated with neovascularization such as vascular endothelial growth factors and subsequently endothelial cell proliferation and migration drives angiogenesis [28].

The ABHD1 gene is located on chromosome 2p23.3 and contains an *α*/*β* hydrolase fold, which is common in hydrolytic enzymes. ABHD1 is expressed in almost all human tissues and especially high in skeletal muscles [29]. And ABHD1 was detected in many cell types such as fibroblasts and endothelial and epithelial. However, the expression of ABHD1 in fibroblasts was markedly lower compared with that in other cell types [29]. Our research mainly discovered the function of ABHD1 in retinal endothelial cells. Our study found that ABHD1 is elevated in both retina tissues of DR patients and HG-induced HRMECs and ABHD1 principally participated in endothelial cell chemotaxis.

Chemotaxis is the phenomenon of cell movement in response to different chemical gradient. Endothelial cell chemotaxis, defined as the directional migration toward chemoattractant gradient, is one of the major mechanisms of endothelial cell migration and typically triggered by growth factors such as VEGF, strongly indicating that ABHD1 might be involved in endothelial dysfunction and neovascularization in DR [30]. In DR, endothelial cells migrate and penetrate the internal limiting membrane toward the vitreous cavity driven by various chemokines, subsequently forming new vessels [31]. Wound healing and CCK-8 experiment of ABHD1-knockout endothelial proved the conjecture and verified that ABHD1 promoted endothelial cell proliferation and migration. Similar to our results, previous research demonstrated that ABHD1 played a role in the proliferation and migration of intestinal stem cells (ISCs) [32].

However, some studies suggest that overexpression of ABHD1 is a protective mechanism in an oxidative stress environment. In D5 dopamine receptor–deficient mice, which presented increased systemic oxidative stress, the mRNA level of ABHD1 upregulated and the overexpression of ABHD1 significantly decreased the total quantity of O2− produced by NADPH oxidase, suggesting that ABHD1 might react to resist oxidative stress condition [18]. Analogously, in an intestinal Notch-activated mouse model, ABHD1 transcript demonstrated significant decline and the Notch signal is known to participate in cell proliferation and apoptosis [33]. Therefore, whether ABHD1 plays a dual role in DR needs to be further explored. Although studies of ABHD1 in diabetes and its complications are rare, research has found that inhibitors of ABHD6 in the same family may play a role in the treatment of type 2 diabetes, insulin resistance, and metabolic syndrome by promoting insulin secretion [34]. Another member of the ABHD family, ABHD5, is also considered as a potential therapeutic target for diabetes, obesity, and cardiovascular diseases [35].

IFs are mainly divided into six types based on their biochemical and structural similarities: keratin, desmin, glial fibrillary acidic protein, vimentin, neurofilament protein, and lamin [36]. IF proteins all have a central *α*-helical rod domain in common which allows self-assembly into oligomers and helps formation into a 10-nm filament [37]. IF proteins are associated with cell migration, proliferation, and angiogenesis [37]. Under hypoxia condition, IF proteins redistribute in endothelial cells and alter in contractility and adhesion [38]. Studies of IF proteins in the retina mainly focused on Müller cells, RPE cells, and glial cells. In the epiretinal membranes of PDR patients, changes in the expression of IF proteins in RPE cells allow the cells to acquire a migratory phenotype of mesenchymal cells [39].

Keratins can be divided into acid cytokeratin (also called Type I cytokeratin) and neutral or basic cytokeratin (also called Type II cytokeratin). A variety of keratins play roles in cell proliferation, cell migration, and tumor angiogenesis. KRT1 is discovered as a novel biomarker in aggressive cancers for its overexpression in epithelial cell surface, and KRT1-targeted reagents could offer innovative therapy for cancer treatments and diagnosis [40]. In psoriasis, aberrant keratin dysregulation may be regulated by VEGF, which is known as a promoter of endothelial cell migration and angiogenesis [41–43]. And ocular anti-VEGF therapy is one of the commonly used standard treatments in DR, giving clues for potential pathogenic effects of ABHD1 in DR [44]. Additionally, in a proteomics analysis of vitreous humor protein in PDR patients, the level of KRT1 in vitreous of PDR patients was significantly increased [45]. KRT2 is a kind of epidermal cytoskeletal protein, and mutations in this gene are associated with bullous congenital ichthyosiform erythroderma [46]. However, research focused on the role of KRT2 in DR is rare. After ABHD1 was knocked down, the expression of KRT1 and KRT2 was significantly decreased in our study. Therefore, we argue that ABHD1 may promote cell proliferation and angiogenesis through the role of keratin.

Collectively, our study is the first to find that ABHD1 is elevated in DR and that ABHD1 might facilitate IF-mediated endothelial cell chemotaxis by KRT1 and KRT2, potentially instructing new therapies for DR. Further research on the mechanism of ABHD1 in DR is needed.

There are still some limitations in our research. First, in addition to validation in the human retina database and endothelial cell lines, we will further refine the in vivo experiments, carry out ABHD1-specific gene knockout in mice, and further construct models of hyperglycemia and hypoxia to simulate diabetic status and then observe the effect of ABHD1 knockout on DR fundus findings such as retinal vascular leakage and neovascularization. In consideration of the role of ABHD1 in lipid metabolism, deeper exploration of how ABHD1 regulates lipid metabolism disorders especially in diabetes status will extend in the future.

## Figures and Tables

**Table 1:** Primer sequences of real-time quantitative PCR.

**Gene**	**Forward primer**	**Reverse primer**
ABHD1	CTCACTGCTCTTCAATCAGCCC	GCGCTCATCAAACTGGCGGATT
KRT1	CAGCA TCATTGCTGAGGTCAAGG	CATGTCTGCCAGCAGTGATCTG
KRT2	ACCTACCGCAAACTGCTGGAGG	CAGAACCTCCAAAGGCAGCCTT
18S rRNA	GTAACCCGTTGAACCCCATT	CCATCCAATCGGTAGTAGCG

## Data Availability

The data that support the findings of this study are available from the corresponding authors upon reasonable request.
